# An Investigation of Neurochemical Changes in Chronic Cannabis Users

**DOI:** 10.3389/fnhum.2019.00318

**Published:** 2019-09-19

**Authors:** Sharlene D. Newman, Hu Cheng, Ashley Schnakenberg Martin, Ulrike Dydak, Shalmali Dharmadhikari, William Hetrick, Brian O’Donnell

**Affiliations:** ^1^Department of Psychological and Brain Sciences, Indiana University, Bloomington, IN, United States; ^2^Program in Neuroscience, Indiana University, Bloomington, IN, United States; ^3^School of Health Sciences, Purdue University, West Lafayette, IN, United States; ^4^Department of Radiology and Imaging Sciences, Indiana University School of Medicine, Indianapolis, IN, United States

**Keywords:** cannabis, magnetic resonance spectroscopy, creatine, glutamate, anterior cingualte cortex

## Abstract

With the legalization of recreational cannabis (CB) the characterization of how it may impact brain chemistry is essential. Magnetic resonance spectroscopy (MRS) was used to examine neurometabolite concentrations in the dorsal anterior cingulate (dACC) in chronic CB users (*N* = 26; 10 females) and controls (*N* = 24; 10 females). The concentrations of glutamate (Glu), total creatine (tCr), choline (Cho), total *N*-acetylaspartate (tNAA), and myo-inositol (mI) were estimated using LCModel. The ANCOVAs failed to show significant differences between controls and CB users. Regression analyses were then performed on the CB group to model each neurometabolite to determine its relationship to monthly CB use, sex, the interaction between CB use and sex. tCr was found to be predicted by both monthly CB use and sex. While the regression model was not significant the relationship between monthly CB use and Glu appears to be modulated by sex with the effect of monthly use (dose) being stronger in males. tNAA failed to show an effect of CB use but did reveal an effect of sex with females showing larger tNAA levels. Although the results presented are preliminary due to the small sample size they do guide future research. The results presented provide direction for further studies as they suggest that dose may significantly influence the observance of CB effects and that those effects may be modulated by sex. Studies with significantly larger sample sizes designed specifically to examine individuals with varying usage as well as sex effects are necessary.

## Introduction

The use of cannabis (CB) has increased over the past decade in the United States, and past-year prevalence of CB use exceeds 10% (Grucza et al., 2016) with few users seeking treatment (Brown et al., 2003). However, delta-9-tetrahydrocannabinol (THC), the compound responsible for the psychoactive effects of CB, has been found to alter neurochemistry (Sneider et al., 2013; Colizzi et al., 2016) which may interact with the development of psychiatric disorders such as schizophrenia and depression (Auer et al., 2012; Egerton and Stone, 2012). In terms of the impact on neurochemistry, the few studies using magnetic resonance spectroscopy (MRS) to measure neurometabolites in humans have reported CB related modulations in glutamate (Glu), creatine (Cr), *N*-acetylaspartate (NAA), myo-Inositol (mI) and choline (Cho) (Sneider et al., 2013).

CB use impacts an array of neurochemicals with human studies reporting that CB exposure interacts with NAA, Cr, mI and Cho in addition to Glu (Cowan et al., 2009; Sneider et al., 2013; Bitter et al., 2014). For example, in a review Sneider et al. (2013) found that CB users had lower NAA (found in 6 out of 8 studies) than did controls. They also reported that frequency or duration of CB use was associated with lower levels of NAA, Cho and mI. NAA is an indicator of neuronal health (Chawla et al., 2014); therefore, the lower levels of NAA suggest that CB use may have a toxic effect on neurons. Additionally, Hermann et al. (2007) found that recreational male CB users had lower NAA/tCr than control non-users and Yücel et al. (2016) found lower levels of NAA in the hippocampus in CB users. It should be noted that the reports reviewed measured NAA from different brain regions and often reported NAA as a ratio making replication studies important.

As mentioned, CB use, particularly heavy use, has been linked to psychiatric disorders (Moore et al., 2007; Lev-Ran et al., 2014). A meta- analysis of longitudinal studies examining the relationship between CB use and depression found a moderate association between heavy CB use (defined as at least weekly use) and increased risk of developing depression (Lev-Ran et al., 2014). Additionally, a recent study using genome-wide data from the International Cannabis Consortium and the Psychiatric Genomics Consortium (Gage et al., 2017) found a small causal effect of CB use on the development of schizophrenia and a large effect of the reverse – schizophrenia risk predicts CB use. Regardless of the direction of causation, there is a clear relationship between CB use and psychosis.

Neurochemistry may be the key to understanding the relationship between CB use and psychiatric disorders. In a recent review it was found that NAA, Glu and Cr were systematically found to be altered in psychosis patients (Li et al., 2018). Creatine which plays a role in regulating energy metabolism as a neuromodulator has been linked to psychiatric disorders including schizophrenia (Volz et al., 1998; Allen, 2012) and mood disorders (depression and anxiety) (Agren and Niklasson, 1988; Coplan et al., 2006; Mirza et al., 2006; Allen, 2012). NAA is linked to neuronal integrity and mitochondrial dysfunction (Moffett et al., 2007; Larabi et al., 2017). Li et al. (2018) also found in their review that NAA appears to be downregulated in psychosis which they argue is consistent with studies suggesting myelination abnormalities in psychosis (Flynn et al., 2003; Mighdoll et al., 2015). Finally, Glu is the most abundant excitatory neurotransmitter in the brain and has also been linked to psychiatric disorders. For example, a recent study found that ACC Glu levels were higher in symptomatic compared to remitted schizophrenia patients (Egerton and Stone, 2012) while reductions in Glu were found in the ACC of patients with major depression (Auer et al., 2012). In a recent study by Rigucci et al. (2018) examining prefrontal Glu in early psychosis patients who use CB and non-CB users found that Glu was lower in early psychosis users compared to both controls and early psychosis non-users but there were no differences between the non-user early psychosis group and controls. However, a greater decline in Glu with age was found in the early psychosis users compared to the two non-user groups suggesting that CB use may interact with disease progression. In sum, given that CB use has been found previously to be correlated with changes in NAA, Cr and Glu levels and these same neurochemicals are linked to psychiatric disorders, disorders that have also been associated with CB use, it is important to further explore these relationships.

The primary aim of the current study was to examine the relationship between chronic CB use and neurochemistry in humans using MRS. The target of investigation was the dorsal anterior cingulate (dACC) cortex. The ACC has also been found to have high CB1 receptor density (Glass et al., 1997; Tsou et al., 1998) suggesting that CB is likely to have an impact on the processing and neurochemistry of the region. It should also be noted that the ACC is a heterogeneous region with a number of subregions that have different cytoarchitecture and connectivity patterns. The current study focuses on the dACC which has been linked to inhibitory control processes and has been shown previously to have Glu concentration differences in CB users (Prescot et al., 2011, 2013). Additionally, because the region has been examined previously it allows for extension and replication of previous studies.

Differential effects of CB use as a function of sex have been reported previously in humans as well as in animal models (Calakos et al., 2017). For example, male CB users exhibit higher circulating levels of delta9-tetrahydrocannabinol (THC), the psychoactive component of CB (Jones et al., 2013); show larger cardiovascular and subjective effects than female users (Leatherdale et al., 2007); display more withdrawal symptoms and are less likely to be CB-only users (Hasin et al., 2008). Preclinical studies in rats have found that males are more sensitive to the hyperphagic and hypophagic effects of the CB1 receptor agonists and antagonists, respectively (Diaz et al., 2009) and to their hypothermic and hyperthermic effects (Farhang et al., 2009); females show greater catalepsy, antinociception and locomotor effects (Tseng and Craft, 2004); and decreases in both exploratory behavior and emotionality/anxiety levels (Biscaia et al., 2003). The previous research strongly suggests that females are different from males in their response to cannabinoids. However, there are very few studies examining neurochemical sex differences in humans. Those few studies that examine effects of sex show sex differences. For example, a study examining sex differences in CB users as a secondary aim found that female users had higher levels of mI and lower levels of Glu + glutamine (Glx) in the dorsal striatum than control females, while male users failed to show any effect (Muetzel et al., 2013). Also, Wiers et al. (2016) using PET found that frontal dopamine signaling is impaired in female CB users but not males. A secondary analysis performed in the current study was designed to examine the interaction between sex and CB use on neurochemistry. It was predicted that CB use has a greater impact on female users than male users.

It should be noted that differences in neurochemistry between CB users and non-users in humans have not been consistently observed (Cousijn et al., 2018). For example, in the Sneider et al. (2013) review two of the 8 studies failed to show an effect of CB use on NAA. There are a number of potential explanations for the discrepant findings including that the effects of CB use may be dependent upon age of participants, duration of use, and brain region examined. An additional explanation for discrepant findings is the variation in the definition of chronic CB use. Currently there is no consistency across studies regarding the CB use criterion within the chronic CB user group [e.g., 10 uses in past 12 months (Wright et al., 2016) to 5 times a week in the past 12 months (Muetzel et al., 2013)]. In the current study we examined whether CB dosage, defined here as monthly instances of use, predicts neurometabolite levels and hypothesized that higher CB use will be correlated with neurometabolite levels.

## Materials and Methods

### Participants

A total of 69 current users and non-users participated in the study. Subjects were recruited by local advertisements. After detailed description of the study, written and verbal informed consent was obtained from each participant. Subjects were asked to refrain from alcohol or CB use the day prior to the MRI scan. This study was carried out in accordance with the recommendations of and approved by Indiana University’s Institutional Review Board for the protection of human subjects. All subjects gave written informed consent in accordance with the Declaration of Helsinki.

The exclusion criteria include: younger than 18 years or older than 40; presence of any neurological disorder; history of head trauma with loss of consciousness greater than 10 min; learning disability; diagnosed psychological disorders including major depression, panic disorder, or psychosis; use of illicit drugs (other than CB); alcohol dependence; and contraindication to MRI. For the CB user group an additional exclusion criteria was CB use less than one instance per week.

Participants completed a battery of assessments including the Structured Clinical Interview for DSM-IV-TR (SCID-IV-TR), Research Version (First et al., 2002); a written drug use questionnaire; a 6-month time line follow back assessment to estimate current and past use of CB and alcohol; the short Michigan alcohol screening test (SMAST); Fagerstrom Test for Nicotine Dependence (FTND); and the Wechsler Abbreviated Scale of Intelligence (WASI; Wechsler, 1999). The control subjects had no history of substance dependence, a negative urine screen for CB and other substances, and no use of CB in the past 3 months. Groups did not significantly differ in age, IQ score, sex, days since last alcohol use at the time of screening, or drinks per week (*p* > 0.1). Additionally, when examining just the CB group, there were no sex differences in age, age of CB use onset, monthly CB use, or lifetime CB use (*p* > 0.1); females were similar to males. CB use disorder was not a requirement for the CB user group.^1^

### MRI Acquisition

Image acquisition was performed on a 3T Siemens Tim-Trio MRI scanner. Foam pads were used to minimize head motion for all participants. High-resolution T1-weighted anatomical images were acquired in the sagittal plane using an MP-RAGE sequence (TR = 1.8 s; TE = 2. 67 ms; inversion time = 0.9 s; flip angle 9°; imaging matrix = 256 × 256; 192 slices; voxel size = 1 × 1 × 1 mm^3^). MRS was performed using a single-voxel PRESS sequence (TR/TE = 2000/30 ms, bandwidth = 2000 Hz, 2048 data points, 120 averages, scan time = 4 min), followed by a water reference scan (8 averages). Each voxel measurement began with the FASTMAP shimming method twice (Gruetter, 1993; Gruetter and Tkác, 2000). FASTMAP is a pulse sequence that samples the magnetic field along a group of radial columns and then adjusts the first order and second order shims. Each run of FASTMAP is one iteration. Manual shimming was performed only if FASTMAP did not give a good shimming result. The full width at half maximum (FWHM) of the linewidths of the water peak was all below 14 Hz after these procedures. All scans were visually checked to ensure acceptable MRI quality.

### Voxel Placement

**FIGURE 1:**
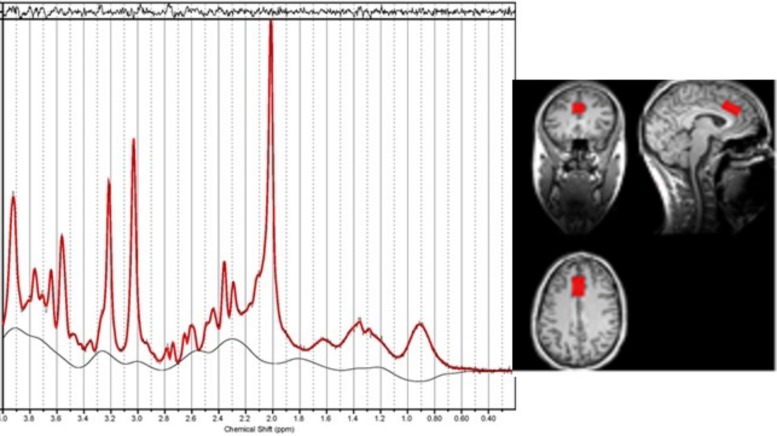
An example of the location of the voxel for Magnetic resonance spectroscopy (MRS) in the dorsal anterior cingulate along with the resultant spectrum processed by LCModel. The fitted spectrum (red) is superimposed on the original spectrum (black); the residual of fitting is on the top while the baseline is at the bottom. The linewidth is 0.033 ppm.

The MR spectroscopy voxel was positioned in the dACC using the T1-weighted image. The voxel was positioned in the following way: locate the mid-slice of the corpus callosum on the sagittal slice, then place the voxel directly above the superior and posterior genu of the corpus callosum with the long axis aligned with them (see Figure 1). The voxel size was 15 × 20 × 25 mm^3^.

### MRS Analysis

The MRS data were processed with LCModel (version 6.2-0R)^2^ using default settings for water attenuation, estimated water concentration and baseline modeling. LCModel was used to fit each spectrum as a weighted linear combination of a basis set of *in vitro* spectra from individual metabolite solutions. The basis set was provided by LCModel for TE 30 ms and 123 MHz. The water reference signal was used for eddy current correction and scaling the metabolite concentrations. The concentrations of glutamate (Glu), total creatine (tCr), choline (Cho), total *N*-acetylaspartate (tNAA) and myo-inositol (mI) were expressed in institutional units. LCModel also reports an estimated relative standard deviation (%SD) for each fitted component, which is equivalent to the Crame’r-Rao lower bounds (CRLB). Subjects were excluded if the sum of CRLB values of creatine and phosphocreatine was greater than 17%. This threshold was chosen based on the visual check of spectrum quality. It is stricter than that used in the previous literature, which was normally set to 20% for any individual metabolite. As a matter of fact, the CRLB values were all smaller than 20% for Glu and other metabolites for the remaining subjects in our study.

The neurometabolite concentrations were normalized using a method described by Gussew et al. (2012). This method controls for MRS signal differences in tissue composition within the measured voxel across subjects. The high-resolution structural scan acquired to position the voxel during data acquisition was used to determine the tissue composition. The T1-weighted image was segmented for gray matter, white matter, and CSF with SPM12^3^. The corresponding fraction of tissue volumes in the MRS voxel was calculated and used to correct for neurometabolite concentration with respect to heterogeneous tissue compositions according to equation 2 in the paper by Gussew et al. (2012). Additional parameters for the correction included the T1 and T2 relaxation time of water in GM (1.82/0.10 s), WM (1.08/0.07 s), and CSF (4.16/0.50 s) (Lin et al., 2001; Stanisz et al., 2005; Piechnik et al., 2009), relative water contents in GM (0.78), WM (0.65) and CSF (1.0) (Ernst et al., 1993), and T1 and T2 of Glu in the GM (1.27/0.16 s) and WM (1.17/0.17) (Ernst et al., 1993; Mlynárik et al., 2001), respectively. Thus, corrected metabolite concentrations are given in institutional units. Because tCr was found to be predicted by CB use we did not normalize other metabolites to tCr. An analysis examining the ratio of neurometabolites with tCr was performed to make comparisons with previous studies easier. Those results can be found in the Supplementary Table S7 and Supplementary Figure S2.

### Statistical Analyses

A correlation analysis was performed to explore the relationship between measures. Secondly, a 2 (group) by 2 (sex) ANOVA was performed on each MRS measure to examine group and sex effects. Finally, a two-step multiple regression analysis was performed with only the CB users to determine the impact of monthly use on metabolite measures. In the first step monthly use, and sex were entered into the model. In the second step the interaction between sex and monthly CB use was included (an analysis with alcohol and nicotine use measures entered in the model can be found in the Supplementary Material). Analyses were performed using SAS version 9.4. Multiple comparison correction was performed using Bonferroni correction. For the model statistics an alpha of 0.025 (0.05/2) and for the parameter estimates an alpha of 0.05/#of predictors were used to determine significance.

## Results

Of the 69 participants, six were removed due to a history of alcohol use disorder, 4 were removed due to an axis I psychiatric disorder, 2 due to insufficient CB use, 5 due to noisy MRS data, and 2 due to neurological disorders. Data from fifty participants were included in the final analyses – twenty-six current (CB) users and 24 healthy non-user controls (see Table 1).

**TABLE 1:** Demographics.

	**Controls**	**CB Users**
*N*	24	26
#Males	10	10
Age	21.5 ± 2.3	21.4 ± 4.5
	(18−26 years)	(18−39 years)
Age of CB initiation	n/a	16.4 ± 2.5 years
Average monthly CB use		33.1 ± 27.2 instances/month^∗^
	0	19.7 ± 9.1 days/month^∗^
Lifetime CB use (instances)	1 ± 2.7	1442.1 ± 2115.5^∗^
Average days since last CB use (prior to scan)	n/a	1 ± 1.8 days
Average days since last alcohol use (prior to scan)	138.9 ± 403.2	22.2 ± 59.4
Average drinks per week	2.2 ± 2.9	3.3 ± 3.2
FNTD	0 ± 0	0.77 ± 0.27
% have used nicotine in month prior to scan	4%	19%
WASI	113.7 ± 11.1	110.7 ± 9.2

*^∗^ indicates significant difference between CB users and controls, based on 2-tailed *t*-test.*

### Voxel Tissue Composition

The majority of the MRS voxel was composed of gray matter in both groups. An independent samples *t*-test was performed and the gray matter concentration did not differ between groups (*p* = 0.75; control group 89% gray matter; user group 85% gray matter). White matter concentration was found to be different between groups with the user group having a larger concentration of white matter (*p* = 0.04). The tissue fractions were then used to correct for the concentrations as indicated by Gussew et al. (2012). The analysis was also performed with the ratio of GM/WM included as a covariate (see Supplementary Table S8).

### Data Quality

The FWHM and S/R from the LCModel Miscellaneous Output are measures of the linewidth and signal-to-noise ratio (SNR) of the *in vivo* spectra. Independent samples *t*-tests were used to examine measures of data quality. No differences were found between the user and control groups in linewidth (*p* = 0.44; control: 0.0347 ± 0.0042; CB: 0.0335 ± 0.006) or SNR (*p* = 0.63; control: 63.8 ± 10.1; CB: 62.6 ± 7.5).

### Correlation Analyses

The correlation results are shown in Tables 2, 3. As shown, the CB user group shows significant positive correlations between tNAA and tCr, Glu and mI while the control group does not show such significant correlations between those metabolites. Additionally, in the CB user group there is a negative correlation between monthly CB use and drinks per week such that those who drink more use CB less.

**TABLE 2:** Correlation analysis for control group.

	**CBmonth**	**Drinks**	**FNTD**	**tCr**	**Glu**	**tNAA**	**mI**	**Cho**
CBmonth	.	.	.	.	.	.	.	.
drinks		1	.	0.24	0.04	–0.17	0.14	0.04
FNTD				.	.	.	.	.
tCr				1	0.02	0.18	**0.45**	0.39
Glu					1	–0.3	0.14	–0.3
tNAA						1	0.04	0.09
mI							1	**0.55**
Cho								1

*Bolded values indicate statistical significance.*

**TABLE 3:** Correlation analysis for CB user group.

	**CBmonth**	**Drinks**	**FNTD**	**tCr**	**Glu**	**tNAA**	**mI**	**Cho**
CBmonth	1	−**0.55**	–0.26	**0.47**	0.25	0.34	0.28	0.09
drinks		1	–0.17	–0.12	–0.23	0.02	–0.13	–0.16
FNTD			1	–0.21	–0.04	–0.36	0.16	–0.005
tCr				1	0.38	**0.66**	**0.4**	0.29
Glu					1	**0.46**	**0.49**	–0.09
tNAA						1	**0.61**	0.22
mI							1	0.38
Cho								1

*Bolded values indicate statistical significance.*

#### TCr

**FIGURE 2:**
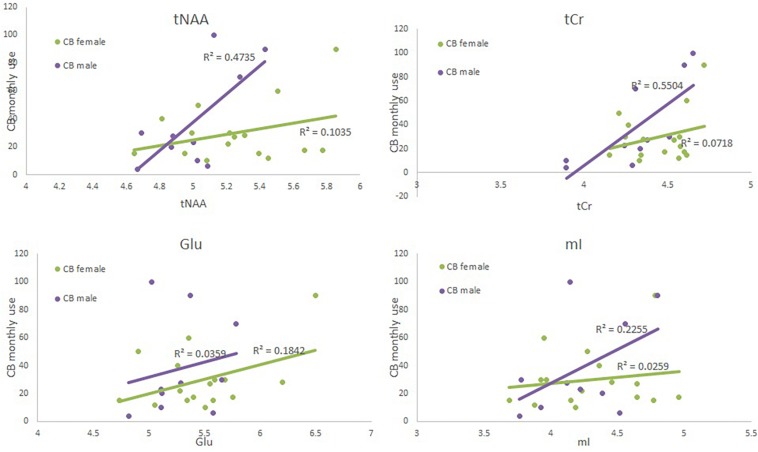
Scatter plots depicting each neurometabolite level (*x*-axis), monthly CB use (*y*-axis) and sex (green = CB user females, and purple = CB user male4).

The ANOVA failed to show an effect of group or sex (*F* < 1); additionally the interaction was also not significant [*F*(1,49) = 1.87, *p* = 0.18]. Both regression models were significant (see Table 4 and Figure 2). CB monthly use significantly predicted tCr levels in both the model with and without the interaction term. Sex was marginally significant in the model without the interaction term but significant in the model with the term.

**TABLE 4:** Regression analysis with tCr as the dependent variable for the CB users only.

**Variable**	**DF**	**Parameter**	**Standard**	***t***	**Pr > |*t*|**	**Standardized**	**Variance**	**95% Confidence**	
		**estimate**	**error**			**estimate**	**inflation**	**limits**	
***Without interaction term: F(2,26) = *7***.**16, p = *0***.**0036, R^2^ = *0***.**37, Bayes Factor = *6***.**58***									
Sex	1	–0.16	0.072	–2.32	***0.029***	–0.38	1.01	–0.32	–0.018
CBmonth	1	0.0037	0.0011	3.23	**0.0035**	0.53	1.01	0.0013	0.006
***With the interaction term: F(3,23) = *5***.**43, p = *0***.**0057, R^2^ = *0***.**4145, Bayes Factor = *3***.**1***									
Sex	1	–0.22	0.83	–2.66	**0.014**	–0.50	1.36	–0.39	–0.049
CBmonth	1	0.003	0.0013	2.42	***0.024***	0.43	1.24	0.00043	0.0056
CBmonth^∗^Sex	1	0.0034	0.0027	1.27	0.22	0.26	1.64	–0.0022	0.009

*Bolded values indicate statistical significance.*

#### Glu

The ANOVA failed to show an effect of group or sex (*F* < 1); additionally the interaction was also not significant [*F*(1,49) = 2.53, *p* = 0.12]. Both regression failed to reach significance (see Table 5 and Figure 2). When examining each predictor variable the parameter estimate for monthly use appears to be modulated by the introduction of the interaction term to the model (although the factor is not significant when corrected for multiple comparisons) suggesting that the effect of monthly CB use is different for males and females. However, these results should be interpreted with great caution given the small sample size and small effect size.

**TABLE 5:** Regression analysis with Glu as the dependent variable for the CB users only.

**Variable**	**DF**	**Parameter**	**Standard**	***t***	**Pr > |*t*|**	**Standardized**	**Variance**	**95% Confidence**	
		**estimate**	**error**			**estimate**	**inflation**	**limits**	
***Without interaction term: F(2,24) = *2***.**31, p = *0***.**12, R^2^ = *0***.**16, Bayes Factor = *0***.**21***									
Sex	1	–0.23	0.15	–1.49	0.15	–0.28	1.0	–0.54	0.087
CBmonth	1	0.0041	0.0024	1.7	0.1	0.32	1.0	–0.00087	0.009
***With the interaction term: F(3,23) = *2***.**08, p = *0***.**13, R^2^ = *0***.**21, Bayes Factor = *0***.**12***									
Sex	1	–0.12	0.18	–0.67	0.51	–0.15	1.36	–0.48	0.24
CBmonth	1	0.0055	0.0026	2.08	***0.048***	0.43	1.24	0.000043	0.011
Sex^∗^CBmonth	1	–0.007	0.0057	–1.23	0.23	–0.29	1.64	–0.019	0.0048

*Bolded values indicate statistical significance.*

#### tNAA

The ANOVA failed to show an effect of group or an interaction between group and sex (*F* < 1). However, there was a significant effect of sex [*F*(1,49) = 7.44, *p* = 0.009]; females had a higher level of tNAA than did males. Neither regression model was significant (see Supplementary Table S5, Supplementary Figure S1, and Figure 2). When examining the predictor variables sex approached significance.

#### mI

The ANOVA failed to show an effect of group or sex (*F* < 1); additionally the interaction was also not significant [*F*(1,49) = 2.7, *p* = 0.11]. Neither regression model was significant (see Supplementary Tables S1, S4, Supplementary Figure S1, and Figure 2).

#### Cho

The ANOVA failed to show an effect of group [*F*(1,49) = 2.12, *p* = 0.15], sex (*F* < 1), or an interaction [*F*(1,49) = 1.01, *p* = 0.32]. Neither regression model was significant (see Supplementary Table S6 and Supplementary Figure S1).

## Discussion

The goal of the current study was to examine the relationship between chronic CB use and neurochemistry in humans. Neurometabolite concentrations in the dACC were measured using MRS. Unlike in some previous studies, the current study failed to show significant differences between the control and CB user group. However, when using regression models to examine the factors that may contribute to the variance in neurometabolite concentrations within the CB user group two major observations were reported. First, monthly CB use consistently predicted total creatine in the CB user group regardless of the other factors entered into the regression model. Second, sex was a consistent predictor of total NAA in the CB group and it was a significant factor in the ANOVA.

Total creatine is considered to have stable concentrations and, as mentioned above, has been widely used as an internal reference such that many MRS studies report concentrations of other metabolites as a ratio of tCr. In the current study, tCr was consistently found to be predicted by monthly CB use regardless of the other measures included in the regression model. The finding that tCr is modulated by CB use has been reported previously (Prescot et al., 2011). Prescot et al. (2011) found that tCr levels decreased in adolescent CB users compared to controls. As a result of this modulation of tCr by CB use the measures presented in the current study were not normalized to it and instead all measures were normalized to tissue water and corrected for tissue composition.

Recently it has been reported that Cr has neuroprotective properties with it potentially being used to treat a number of disorders. For example, Cr was given to children and adolescents with traumatic brain injury and was shown to improve cognitive performance (Sakellaris et al., 2006). Creatine kinase and its substrates creatine and phosphocreatine are part of the cellular energy buffering and transport system that connects sites of energy production (mitochondria) to sites of energy consumption (Hemmer and Wallimann, 1993). Previous studies have found that Cr administration increases brain concentrations of phosphocreatine and inhibits mitochondrial permeability transition, both of which may exert neuroprotective effects (Hemmer and Wallimann, 1993; O’Gorman et al., 1996; Ferrante et al., 2000). Phosphocreatine has also been found to stimulate synaptic Glu uptake, reducing extracellular Glu (Xu et al., 1996), thereby providing an additional neuroprotective pathway. Another potential neuroprotective mechanism of Cr is related to its relationship with NAA. Ferrante et al. (2000) found a correlation between Cr and NAA in Cr treated transgenic Huntington’s mice but not untreated mice. NAA has been shown previously to be an indicator of neuronal health (Chawla et al., 2014). Interestingly, in the current study a positive correlation between tNAA and tCr was found for the CB users (*r* = 0.66) but not the controls (*r* = 0.18). Although the variance in both tNAA and tCr in the CB user group can be explained partially by CB monthly use, the correlation between tNAA and tCr remains significant when partialing out the impact of monthly CB use (*r* = 0.6, *p* = 0.001). These results suggest that the young adult users examined in this study may have increased brain concentrations of tCr as a mechanism to protect itself from damage caused by an increase in exogenous cannabinoids. This is different from the results reported by Prescot et al. (2011) which shows that adolescents show decreases in tCr. This discrepancy may be due to differences in the subject population. The population examined in this study is a high functioning chronic CB user group with normal to high IQ. Further studies examining how age of CB initiation, cognitive capacity and years of use may interact with tCr are necessary.

A second finding of the study is a sex differences in tNAA levels such that women had a higher level of tNAA than men. While there are few studies examining sex differences in tNAA one recent study reported similar results. Silaidos et al. (2018) examined NAA as a proxy for mitochondrial dysfunction. There they found that female participants had higher NAA levels in both gray and white matter than male participants did; a similar finding to that reported in the current study. While there was no interaction between sex and CB use for the NAA measure, this sex difference and how it may interact with the effects of CB use warrants further study.

Glutamate is one of the brain’s primary excitatory neurotransmitters whose concentration is tightly controlled due to its potential toxic properties. Although previous studies have found a relationship between CB use and Glu the current study failed to show a strong effect. However, the results do advocate for future studies with a much larger sample size in order to fully explore factors that may interact with the relationship between CB use and Glu levels. For example, the current results show that monthly CB use begins to approach significance when the interaction between monthly use and sex is included in the model suggesting that Glu levels may be dependent on the amount of CB use and that the relationship between Glu and CB use may be modulated by sex. The potential influence of sex on the relationship between CB dose and Glu levels support previous reports in preclinical studies and previous studies in humans by Muetzel et al. (2013) and Prescot et al. (2011). In fact, Prescot et al. (2011) had a very similar result in that the effect of Glu was increased when sex was included in the model. Again, while the interpretation of this effect should be considered with caution, they clearly indicate a direction for future research.

In addition, more fully examining the interaction between CB use and other substance use including alcohol and nicotine on neurometabolite levels is important. The current study attempted to control for the use of other substances, however, both alcohol and nicotine are used in higher rates in the CB user population than the non-user in the current study. Additionally, a recent study by Schulte et al. (2017) found that differences in dACC Glx (glutamate + glutamine) were not dependent on the type of substance used whether it be nicotine or polysubstance users.

It should be noted that our results are contradictory to those reported previously by Prescot et al. (2011) and Muetzel et al. (2013) in that both of these previous studies reported a decrease in neurometabolite levels in the ACC of adolescents and in the striatum of college-aged individuals, respectively, while we show increases in college-aged individuals. There are a number of reasons for these discrepant results. First, most studies, including Muetzel et al. (2013), use tCr to normalize neurometabolite concentrations and report a ratio with tCr. Because we observed CB effects of tCr we do not report concentrations in terms of a ratio. Second, in the current study we normalized the differing effects of gray and white matter on the MRS signal which was not performed in the previous studies. Finally, there were differences in the LCModel processing and the version of the software used to perform quantification. For instance, the analysis window was set to 0.2–4.0 ppm in our study in contrast to 0.5–4.5 ppm by Prescot et al. (2011, 2013). The latter three reasons may account for different results even using the ratio to tCr (Supplementary Table S7 and Supplementary Figure S2). These differences in the analysis makes direct comparison across studies difficult. Even with these differences, the relationship between CB use and sex are very similar across studies.

### Limitations

The results presented should be interpreted with caution. There were some limitations regarding the participants. The number of participants, while larger than some previous studies, is rather small, particularly when examining the effect of current CB use and sex. In addition, the results of the current study suggest that a larger sample size with a range of CB use levels (dosage) as well as better characterization of CB use is necessary to characterize the impact of CB on neurochemistry. Again, although we do not have adequate power to properly address our research questions we do feel that the study is important in that it clearly directs future work and highlights the importance of fully characterizing and controlling factors such as sex and CB dose.

Currently there is no consistency across studies regarding the CB use criterion within the chronic CB user group. The concentration of THC being consumed is not controlled in human studies as it is in preclinical studies making it impossible to control dose. The results reported in the current study demonstrate that the amount of CB use is an important factor to consider when characterizing the impact of CB on neurochemistry. In the current study there was a wide range of monthly CB use with the monthly use having a standard deviation of 27 instances per month. As expected the range of lifetime use is also large with few participants on the far end of the use spectrum. It will be important in future research to ensure an equal distribution of dose in order to examine its effect on neurochemistry.

Another source of variation across studies regarding CB consumption is variability of THC content across geographic regions. The THC products available vary across different regions of the country which likely impact the effects of CB use on neural processing. Unfortunately, we were unable to determine the THC content of the products used by our study participants. However, future studies should consider this issue.

Cannabis use tends to be co-morbid with some psychological disorders like depression and anxiety (Auer et al., 2012) as well as with the use of other drugs like alcohol and nicotine (Blanco et al., 2018). Also, these co-morbidities may also interact with neurochemistry making it difficult to determine the relationship between CB use and brain function, structure and neurochemistry. In the current study we have attempted to control for other substance use and psychological disorders. However, while there are no statistically significant differences between groups it is still possible that they may interact with brain processing differently in the two groups. This requires more extensive research examining poly-substance users as well as those with psychological disorders.

Magnetic resonance spectroscopy is a non-invasive technique that allows for the measurement of a number of molecules including Glu. Glutamate levels in humans have been reliably reported at 3T (Hurd et al., 2004; Cohen-Gilbert et al., 2014; Yasen et al., 2017). While sophisticated 3D MRS sequences are available, single voxel MRS allows for a focus on discreet regions with the higher spatial and spectral resolution necessary for regions with susceptibility issues related to field inhomogeneities like those close to the sinuses (Cohen-Gilbert et al., 2014) (e.g., the nucleus accumbens). The measurement of Glu is complicated by the overlapping resonances of glutamine (Gln). There is some debate as to whether Glu can be reliably separated from Gln at 3T (Mayer and Spielman, 2005; Wijtenburg and Knight-Scott, 2011; Ende, 2015) and it is very likely that our Glu measurements are contaminated by Gln. Also, while MRS technology has advanced to the point that neurometabolites can be reliably measured in humans making it a powerful tool in the study of addiction, the metabolite levels measured by MRS include both intracellular and extracellular components. This is different from methods used in preclinical studies; microlysis in animal studies primarily measure extracellular concentrations. This difference in measures makes the direct comparison to the preclinical literature difficult.

## Conclusion

Cannabis (CB) use is becoming more prevalent with it being legalized for recreational use in a number of states across the United States and countries around the world. Therefore, it is increasingly important to characterize the effect of CB use on brain chemistry, structure and function as it impacts the behavioral and cognitive consequences of use. The current study, even with its limitations, shows that chronic CB use is related to differences in brain chemistry and that those differences may be affected by sex and dose. Understanding these sex differences may be important in the design and implementation of prevention and treatment programs for young users. Additionally, there is the potential to use cannabinoid agonists or antagonists for the treatment of neuropathic pain, glaucoma, multiple sclerosis, migraine, movement disorders and eating/appetite disorders; therefore, understanding the sex differences in cannabinoid pharmacological effects is necessary. Future studies designed to fully characterize the impact of chronic CB use on neurochemistry that accounts for CB dose including THC content, sex, age, age of CB initiation and use of other substances are essential to developing an accurate model of the interaction of CB use and brain chemistry.

## Data Availability

The datasets generated for this study are available on request to the corresponding author.

## Ethics Statement

The research protocol was approved by Indiana University’s Institutional Review Board for the protection of human subjects.

## Author Contributions

SN wrote the manuscript and designed the study. HC was responsible for data analysis and quality. AS was involved in data acquisition, subject recruitment and data management. UD assisted with MRS protocol development and analysis. SD assisted with MRS protocol development. WH contributed to study design. BO’D was involved in the study design.

## Conflict of Interest Statement

The authors declare that the research was conducted in the absence of any commercial or financial relationships that could be construed as a potential conflict of interest.

## Footnotes

1 Based on the SCID 10 of the CB users failed to meet criteria for CUD. In order to meet criteria for diagnosis for abuse/dependence participants had to endorse impairment and/or withdrawal symptoms from use. Given that no collateral reports were obtained and that participants often deny or lack insight about potential impairment we argue for the use of amount of use versus diagnostic categorization. Additionally, when comparing the 2 groups of users – those with and without CUD diagnosis – there were no significant or marginally significant group differences for any of the measures with the exception of age of CB initiation (M = 17 for non-CUD; M = 15 for CUD).

2 http://www.s-provencher.com/

3 http://www.fil.ion.ucl.ac.uk/spm/software/spm12/
