# Supplementary material for: An Investigation of Neurochemical Changes in Chronic Cannabis Users
Source: Front Hum Neurosci. 2019 Sep 19;13:318. doi: 10.3389/fnhum.2019.00318 (PMC6761299; doi:10.3389/fnhum.2019.00318)

**Table S1: Demographics by Gender**

|  | n | Age of CB Initiation | nicotine dependence scale | CB use per month | drinks per week |
| --- | --- | --- | --- | --- | --- |
| female |  |  |  |  |  |
| CB user | 16 | 15.8125 | 0.0625 | 29.9375 | 3.34375 |
| control | 14 | n/a | 0 | 0 | 1.785714286 |
| male |  |  |  |  |  |
| CB user | 10 | 17.3 | 0.1 | 38.05 | 3.27 |
| control | 10 | n/a | 0 | 0 | 2.85 |

**Table S2.** **Regression analysis with tCr as the dependent variable for the CB users only.**

| Variable | DF | Parameter Estimate | Standard Error | t Value | Pr > \|t\| | Standardized Estimate | Variance Inflation | 95% Confidence Limits | |
| --- | --- | --- | --- | --- | --- | --- | --- | --- | --- |
| ***Without interaction term: F(4,22)=3.58, p=0.02, R^2^=0.39, Bayes Factor =0.88*** | | | | | | | | | |
| **Sex** | 1 | -0.1652 | 0.07434 | -2.22 | ***0.0369*** | -0.37258 | 1.02099 | -0.31937 | -0.01102 |
| **CBmonth** | 1 | 0.0039 | 0.00129 | 3.01 | **0.0064** | 0.55556 | 1.23554 | 0.00121 | 0.00658 |
| **drinks** | 1 | 0.00669 | 0.01294 | 0.52 | 0.6103 | 0.09713 | 1.28162 | -0.02014 | 0.03352 |
| **FNTD** | 1 | -0.07192 | 0.13996 | -0.51 | 0.6125 | -0.08961 | 1.10443 | -0.36218 | 0.21834 |
| ***Without interaction term: F(5,21)=3.25, p=0.025, R^2^=0.44, Bayes Factor =0.51*** | | | | | | | | | |
| **Sex** | 1 | -0.21894 | 0.0851 | -2.57 | ***0.0177*** | -0.49379 | 1.37186 | -0.39592 | -0.04197 |
| **CBmonth** | 1 | 0.00323 | 0.00139 | 2.33 | ***0.0301*** | 0.45978 | 1.45463 | 0.000342 | 0.00611 |
| **drinks** | 1 | 0.00699 | 0.01278 | 0.55 | 0.5901 | 0.1015 | 1.28208 | -0.01958 | 0.03356 |
| **FNTD** | 1 | -0.07212 | 0.13821 | -0.52 | 0.6073 | -0.08986 | 1.10443 | -0.35955 | 0.21531 |
| **CBmonth*Sex** | 1 | 0.00344 | 0.00276 | 1.25 | 0.2255 | 0.26212 | 1.64086 | -0.00229 | 0.00918 |

**Table S3.** **Regression analysis with Glu as the dependent variable for the CB users only.**

| Variable | DF | Parameter Estimate | Standard Error | t Value | Pr > \|t\| | Standardized Estimate | Variance Inflation | 95% Confidence Limits | |
| --- | --- | --- | --- | --- | --- | --- | --- | --- | --- |
| ***Without interaction term: F(4,22)=1.3, p=0.37, R^2^=0.17, Bayes Factor = 0.019*** | | | | | | | | | |
| Sex | 1 | -0.22516 | 0.1593 | -1.41 | 0.1715 | -0.27742 | 1.02099 | -0.55553 | 0.10522 |
| CBmonth | 1 | 0.00361 | 0.00277 | 1.3 | 0.2069 | 0.28083 | 1.23554 | -0.00214 | 0.00936 |
| drinks | 1 | -0.01281 | 0.02772 | -0.46 | 0.6487 | -0.10159 | 1.28162 | -0.0703 | 0.04469 |
| FNTD | 1 | -0.00921 | 0.29991 | -0.03 | 0.9758 | -0.00627 | 1.10443 | -0.63118 | 0.61276 |
| ***With the interaction term: F(5,21)=1.2, p=0.34, R^2^=0.22, Bayes Factor =0.013*** | | | | | | | | | |
| Sex | 1 | -0.11461 | 0.18289 | -0.63 | 0.5376 | -0.14121 | 1.37186 | -0.49494 | 0.26572 |
| CBmonth | 1 | 0.00499 | 0.00298 | 1.67 | 0.1089 | 0.38846 | 1.45463 | -0.00121 | 0.01118 |
| drinks | 1 | -0.01343 | 0.02746 | -0.49 | 0.63 | -0.10649 | 1.28208 | -0.07054 | 0.04369 |
| FNTD | 1 | -0.00881 | 0.29703 | -0.03 | 0.9766 | -0.006 | 1.10443 | -0.62652 | 0.6089 |
| Gender_CBmonth | 1 | -0.00708 | 0.00592 | -1.2 | 0.2453 | -0.29455 | 1.64086 | -0.0194 | 0.00524 |

**Table S4.** **Regression analysis with mI as the dependent variable for the CB users only.**

| Variable | DF | Parameter Estimate | Standard Error | t Value | Pr > \|t\| | Standardized Estimate | Variance Inflation | 95% Confidence Limits | |
| --- | --- | --- | --- | --- | --- | --- | --- | --- | --- |
| ***Without interaction term: F(2,24)=1.73, p=0.2, R^2^=0.13, Bayes Factor =0.12*** | | | | | | | | | |
| Sex | 1 | -0.11533 | 0.14108 | -0.82 | 0.4217 | -0.157 | 1.0128 | -0.4065 | 0.17584 |
| CBmonth | 1 | 0.00391 | 0.00223 | 1.75 | 0.0925 | 0.33652 | 1.0128 | -0.0007 | 0.00852 |
| ***With interaction term: F(3,23)=1.15, p=0.35, R^2^=0.13, Bayes Factor =0.033*** | | | | | | | | | |
| Sex | 1 | -0.14331 | 0.16678 | -0.86 | 0.3991 | -0.19508 | 1.36296 | -0.48832 | 0.20171 |
| CBmonth | 1 | 0.00355 | 0.00252 | 1.41 | 0.1711 | 0.30587 | 1.2395 | -0.00165 | 0.00876 |
| Sex*CBmonth | 1 | 0.00179 | 0.00542 | 0.33 | 0.7437 | 0.08242 | 1.6402 | -0.00942 | 0.01301 |
| ***Without interaction term: F(4,22)<1, R^2^=0.13, Bayes Factor =0.011*** | | | | | | | | | |
| Sex | 1 | -0.12031 | 0.14746 | -0.82 | 0.4233 | -0.16377 | 1.02099 | -0.42612 | 0.1855 |
| CBmonth | 1 | 0.00425 | 0.00257 | 1.66 | 0.1117 | 0.36595 | 1.23554 | -0.00107 | 0.00958 |
| drinks | 1 | 0.0069 | 0.02566 | 0.27 | 0.7904 | 0.06051 | 1.28162 | -0.04631 | 0.06012 |
| FNTD | 1 | 0.09361 | 0.27761 | 0.34 | 0.7391 | 0.0704 | 1.10443 | -0.48211 | 0.66934 |
| ***With the interaction term: F(5,21)<1, R^2^=0.14, Bayes Factor =0.004*** | | | | | | | | | |
| Sex | 1 | -0.14859 | 0.17452 | -0.85 | 0.4041 | -0.20228 | 1.37186 | -0.51154 | 0.21435 |
| CBmonth | 1 | 0.0039 | 0.00284 | 1.37 | 0.1847 | 0.33553 | 1.45463 | -0.00201 | 0.00981 |
| drinks | 1 | 0.00706 | 0.02621 | 0.27 | 0.7902 | 0.06189 | 1.28208 | -0.04744 | 0.06156 |
| FNTD | 1 | 0.09351 | 0.28345 | 0.33 | 0.7447 | 0.07033 | 1.10443 | -0.49595 | 0.68298 |
| Sex*CBmonth | 1 | 0.00181 | 0.00565 | 0.32 | 0.7518 | 0.08327 | 1.64086 | -0.00995 | 0.01357 |

**Table S5.** **Regression analysis with tNAA as the dependent variable for the CB users only.**

| Variable | DF | Parameter Estimate | Standard Error | t Value | Pr > \|t\| | Standardized Estimate | Variance Inflation | 95% Confidence Limits | |
| --- | --- | --- | --- | --- | --- | --- | --- | --- | --- |
| ***Without interaction term: F(2,24)=4.2, p=0.027, R^2^=0.26, Bayes Factor =2.28*** | | | | | | | | | |
| Sex | 1 | -0.27778 | 0.11609 | -2.39 | **0.0249** | -0.42292 | 1.0128 | -0.51739 | -0.03818 |
| CBmonth | 1 | 0.00349 | 0.00184 | 1.9 | 0.0695 | 0.33582 | 1.0128 | -0.0003 | 0.00728 |
| ***With interaction term: F(3,23)=2.69, p=0.07, R^2^=0.26, Bayes Factor =0.58*** | | | | | | | | | |
| Sex | 1 | -0.2799 | 0.13757 | -2.03 | *0.0536* | -0.42614 | 1.36296 | -0.56449 | 0.00468 |
| CBmonth | 1 | 0.00346 | 0.00208 | 1.67 | 0.1088 | 0.33322 | 1.2395 | -0.00083 | 0.00776 |
| Sex*CBmonth | 1 | 0.000136 | 0.00447 | 0.03 | 0.976 | 0.00699 | 1.6402 | -0.00911 | 0.00938 |
| ***Without interaction term: F(4,22)=3.1, p=0.036, R^2^=0.36, Bayes Factor =1.0*** | | | | | | | | | |
| Gender | 1 | -0.26512 | 0.11314 | -2.34 | ***0.0286*** | -0.40364 | 1.02099 | -0.49977 | -0.03048 |
| CBmonth | 1 | 0.00354 | 0.00197 | 1.8 | 0.086 | 0.34059 | 1.23554 | -0.00054 | 0.00762 |
| drinks | 1 | 0.00785 | 0.01969 | 0.4 | 0.6941 | 0.0769 | 1.28162 | -0.03299 | 0.04868 |
| FNTD | 1 | -0.34644 | 0.21301 | -1.63 | 0.1181 | -0.29139 | 1.10443 | -0.78819 | 0.0953 |
| ***With interaction term: F(5,21)=2.37, p=0.075, R^2^=0.36, Bayes Factor =0.31*** | | | | | | | | | |
| Gender | 1 | -0.26858 | 0.13423 | -2 | *0.0585* | -0.4089 | 1.37186 | -0.54772 | 0.01057 |
| CBmonth | 1 | 0.0035 | 0.00219 | 1.6 | 0.1248 | 0.33643 | 1.45463 | -0.00105 | 0.00804 |
| drinks | 1 | 0.00787 | 0.02016 | 0.39 | 0.7003 | 0.07709 | 1.28208 | -0.03405 | 0.04978 |
| FNTD | 1 | -0.34646 | 0.21801 | -1.59 | 0.127 | -0.2914 | 1.10443 | -0.79982 | 0.10691 |
| Gender_CBmonth | 1 | 0.000221 | 0.00435 | 0.05 | 0.9599 | 0.01137 | 1.64086 | -0.00882 | 0.00926 |

**Table S6.** **Regression analysis with Cho as the dependent variable for the CB users only.**

| Variable | DF | Parameter Estimate | Standard Error | t Value | Pr > \|t\| | Standardized Estimate | Variance Inflation | 95% Confidence Limits | |
| --- | --- | --- | --- | --- | --- | --- | --- | --- | --- |
| ***Without interaction term: F(2,24)<1, R^2^=0.04, Bayes Factor =0.04*** | | | | | | | | | |
| Sex | 1 | -0.01544 | 0.04259 | -0.36 | 0.7201 | -0.07289 | 1.0128 | -0.10335 | 0.07246 |
| CBmonth | 1 | 0.000675 | 0.000674 | 1 | 0.3263 | 0.20144 | 1.0128 | -0.00072 | 0.00207 |
| ***With interaction term: F(3,23)<1, R^2^=0.05, Bayes Factor =0.013*** | | | | | | | | | |
| Sex | 1 | -0.02606 | 0.05028 | -0.52 | 0.6092 | -0.12299 | 1.36296 | -0.13008 | 0.07796 |
| CBmonth | 1 | 0.00054 | 0.000759 | 0.71 | 0.4836 | 0.16112 | 1.2395 | -0.00103 | 0.00211 |
| Sex*CBmonth | 1 | 0.000681 | 0.00163 | 0.42 | 0.6809 | 0.10843 | 1.6402 | -0.0027 | 0.00406 |
| ***Without interaction term: F(4,22)<1, R^2^=0.084, Bayes Factor =0.005*** | | | | | | | | | |
| Sex | 1 | -0.01882 | 0.04368 | -0.43 | 0.6708 | -0.08881 | 1.02099 | -0.10941 | 0.07177 |
| CBmonth | 1 | 0.000762 | 0.00076 | 1 | 0.327 | 0.22733 | 1.23554 | -0.00081 | 0.00234 |
| drinks | 1 | 0.000672 | 0.0076 | 0.09 | 0.9304 | 0.02041 | 1.28162 | -0.01509 | 0.01644 |
| FNTD | 1 | 0.08055 | 0.08224 | 0.98 | 0.338 | 0.21002 | 1.10443 | -0.09 | 0.2511 |
| ***With interaction term: F(5,21)<1, R^2^=0.091, Bayes Factor =0.002*** | | | | | | | | | |
| Sex | 1 | -0.02931 | 0.05163 | -0.57 | 0.5762 | -0.13834 | 1.37186 | -0.13668 | 0.07805 |
| CBmonth | 1 | 0.000631 | 0.000841 | 0.75 | 0.4615 | 0.18819 | 1.45463 | -0.00112 | 0.00238 |
| drinks | 1 | 0.00073 | 0.00775 | 0.09 | 0.9258 | 0.02219 | 1.28208 | -0.01539 | 0.01685 |
| FNTD | 1 | 0.08052 | 0.08385 | 0.96 | 0.3479 | 0.20992 | 1.10443 | -0.09386 | 0.25489 |
| Sex*CBmonth | 1 | 0.000672 | 0.00167 | 0.4 | 0.6918 | 0.10712 | 1.64086 | -0.00281 | 0.00415 |

**Table S7.** **Regression analysis with MRS measures as a ratio of tCr with monthly use and age of initiation and Sex as predictors for the CB group only.**

| Variable | DF | Parameter Estimate | Standard Error | t Value | Pr > \|t\| | Standardized Estimate | Variance Inflation | 95% Confidence Limits | |
| --- | --- | --- | --- | --- | --- | --- | --- | --- | --- |
| ***Glu/tCr: F(3,23)=0.12, p=0.95*** | | | | | | | | | |
| **CBmonth** | 1 | -0.0001475 | 0.00058107 | -0.25 | 0.8018 | -0.05295 | 1.01512 | -0.00135 | 0.00105 |
| **Age of init** | 1 | -0.00392 | 0.00738 | -0.53 | 0.6001 | -0.11519 | 1.09615 | -0.01919 | 0.01134 |
| **Sex** | 1 | 0.00301 | 0.03841 | 0.08 | 0.9381 | 0.01711 | 1.11000 | -0.07643 | 0.08246 |
| ***tNAA/tCt: F(3,23)=0.45, p=0.72*** | | | | | | | | | |
| **CBmonth** | 1 | -0.0002224 | 0.00037639 | -0.59 | 0.5603 | -0.12070 | 1.01512 | -0.00100 | 0.00055619 |
| **Age of init** | 1 | 0.00319 | 0.00478 | 0.67 | 0.5108 | 0.14175 | 1.09615 | -0.00670 | 0.01308 |
| **Sex** | 1 | -0.01995 | 0.02488 | -0.80 | 0.4308 | -0.17127 | 1.11000 | -0.07141 | 0.03151 |
| ***mI/tCr: F(3,23)=0.31, p=0.82*** | | | | | | | | | |
| **CBmonth** | 1 | 0.00009549 | 0.00049166 | 0.19 | 0.8477 | 0.04000 | 1.01512 | -0.000922 | 0.00111 |
| **Age of init** | 1 | 0.00540 | 0.00624 | 0.86 | 0.3960 | 0.18511 | 1.09615 | -0.00752 | 0.01832 |
| **Sex** | 1 | 0.00339 | 0.03250 | 0.10 | 0.9178 | 0.02247 | 1.11000 | -0.06383 | 0.07061 |
| ***Cho/tCr: F(3,23)=0.09, p=0.96*** | | | | | | | | | |
| **CBmonth** | 1 | -0.0000215 | 0.00015207 | -0.14 | 0.8887 | -0.02954 | 1.01512 | -0.00033609 | 0.00029307 |
| **Age of init** | 1 | -0.0002984 | 0.00193 | -0.15 | 0.8785 | -0.03354 | 1.09615 | -0.00429 | 0.00370 |
| **Sex** | 1 | 0.00520 | 0.01005 | 0.52 | 0.6098 | 0.11300 | 1.11000 | -0.01559 | 0.02599 |

**Table S8.** **Regression analysis for the CB user group including the ratio of GM to WM as a regressor along with gender and monthly CB use.**

| Variable | DF | Parameter Estimate | Standard Error | t Value | Pr > \|t\| | Standardized Estimate | Variance Inflation | 95% Confidence Limits | |
| --- | --- | --- | --- | --- | --- | --- | --- | --- | --- |
| ***Glu: F(3,23)=1.59, p=0.22, R^2^=0.17*** | | | | | | | | | |
| Gender | 1 | -0.21744 | 0.15611 | -1.39 | 0.177 | -0.26792 | 1.02782 | -0.54039 | 0.1055 |
| CBmonth | 1 | 0.00328 | 0.00289 | 1.13 | 0.2684 | 0.25549 | 1.40939 | -0.0027 | 0.00926 |
| ratio | 1 | 0.00481 | 0.00881 | 0.55 | 0.5907 | 0.1222 | 1.39426 | -0.01342 | 0.02304 |
| ***tCr: F(3,23)=4.7, p=0.011, R^2^=0.38*** | | | | | | | | | |
| Gender | 1 | -0.16267 | 0.07381 | -2.2 | **0.0378** | -0.36689 | 1.02782 | -0.31537 | -0.00998 |
| CBmonth | 1 | 0.00334 | 0.00137 | 2.44 | **0.0227** | 0.4761 | 1.40939 | 0.000511 | 0.00617 |
| ratio | 1 | 0.002 | 0.00417 | 0.48 | 0.6362 | 0.09295 | 1.39426 | -0.00662 | 0.01062 |
| ***tNAA: F(3,23)=2.74, p=0.066, R^2^=0.26*** | | | | | | | | | |
| Gender | 1 | -0.28271 | 0.11917 | -2.37 | **0.0264** | -0.43041 | 1.02782 | -0.52922 | -0.03619 |
| CBmonth | 1 | 0.00389 | 0.00221 | 1.76 | 0.0914 | 0.37433 | 1.40939 | -0.00068 | 0.00846 |
| ratio | 1 | -0.0023 | 0.00673 | -0.34 | 0.7357 | -0.07221 | 1.39426 | -0.01622 | 0.01162 |
| ***mI: F(3,23)=1.55, p=0.22, R^2^=0.17*** | | | | | | | | | |
| Gender | 1 | -0.09631 | 0.14142 | -0.68 | 0.5027 | -0.1311 | 1.02782 | -0.38886 | 0.19624 |
| CBmonth | 1 | 0.00236 | 0.00262 | 0.9 | 0.3761 | 0.20346 | 1.40939 | -0.00306 | 0.00778 |
| ratio | 1 | 0.00888 | 0.00798 | 1.11 | 0.2774 | 0.24947 | 1.39426 | -0.00763 | 0.0254 |
| ***Cho: F(3,23)=0.35, p=0.79, R^2^=0.04*** | | | | | | | | | |
| Gender | 1 | -0.01606 | 0.04382 | -0.37 | 0.7173 | -0.07581 | 1.02782 | -0.1067 | 0.07458 |
| CBmonth | 1 | 0.000726 | 0.000812 | 0.89 | 0.3807 | 0.21643 | 1.40939 | -0.00095 | 0.0024 |
| ratio | 1 | -0.00029 | 0.00247 | -0.12 | 0.9081 | -0.0281 | 1.39426 | -0.00541 | 0.00483 |

Figure S1: Mean neurometabolite concentrations for controls and CB users separated by Sex.


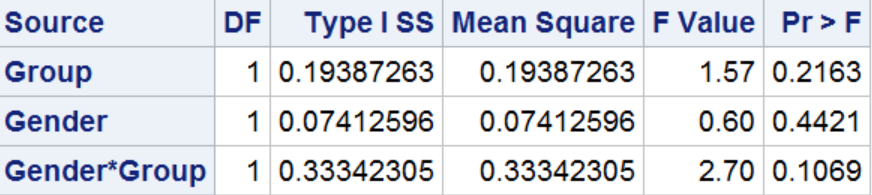

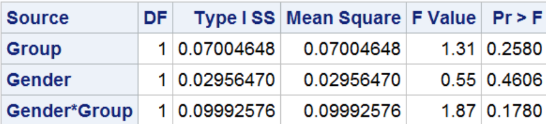

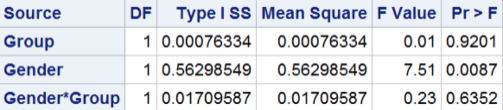

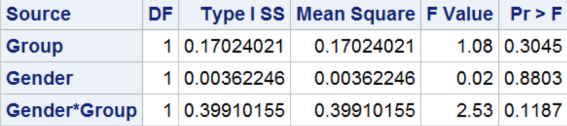

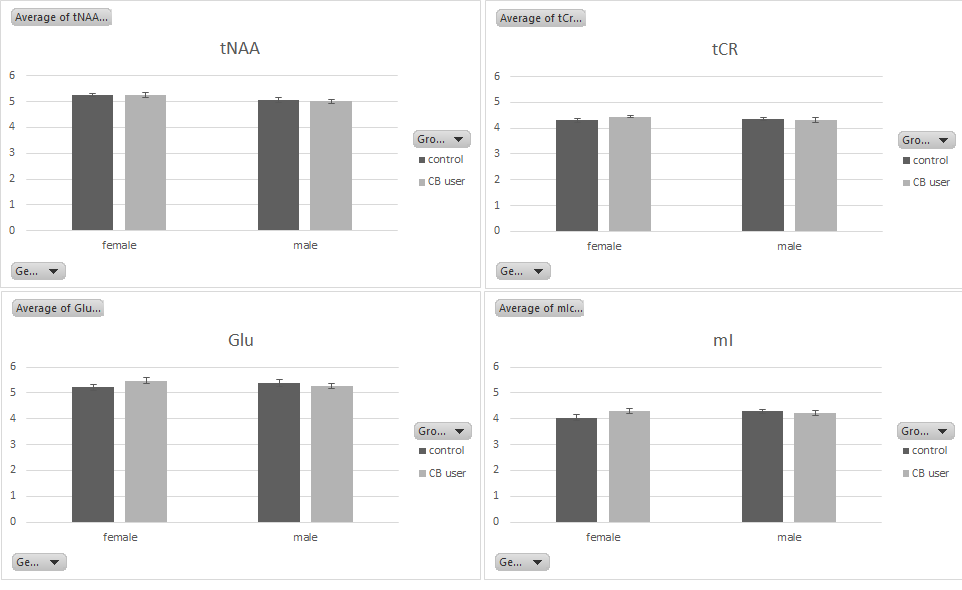


Figure S2: Effect of Group and Sex for the neurometabolites as a ratio of tCr.


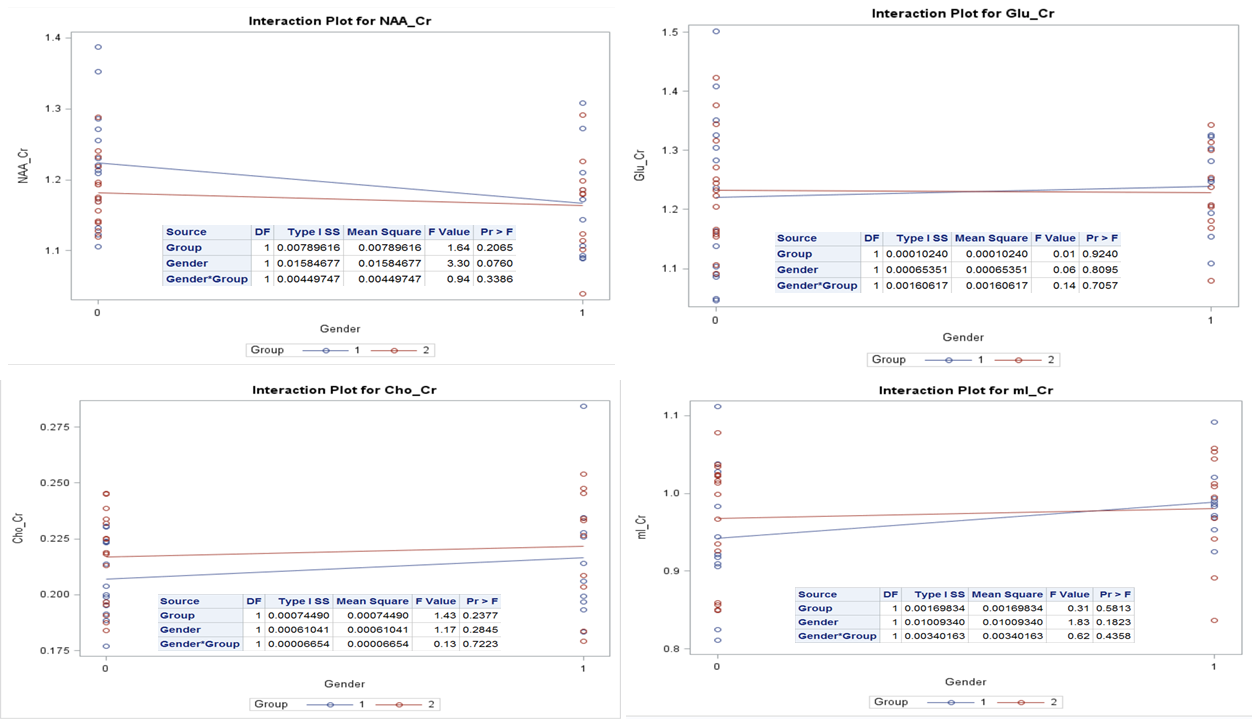

Supplement: Supplementary file 1 [file Data_Sheet_1.docx]
